# A systematic review of cancer risks associated with *MITF* variants

**DOI:** 10.1007/s10689-026-00603-x

**Published:** 2026-09-19

**Authors:** Luigi Monti, Lea Godino, Clio Dessinioti, Alex Stratigos, Olga Papadodima, Alexander Pintzas, Delia Nicoară, Yvette Albert, Stefania Boccia, Sara Barbato, Giulia Erini, Sara Miccoli, Giovanni Innella, Daniela Turchetti

**Affiliations:** 1 Medical Genetics Unit, IRCCS Azienda Ospedaliero-Universitaria di Bologna, Bologna, Italy; 2 1st Department of Dermatology, School of Medicine, Andreas Sygros Hospital, National and Kapodistrian University of Athens, Athens, Greece; 3 Institute of Chemical Biology, National Hellenic Research Foundation, Athens, Greece; 4 The Oncology Institute “Prof.Dr.Ion Chiricuță” Cluj-Napoca (IOCN), Cluj-Napoca, Romania; 5 IRCCS, Fondazione Policlinico Universitario A. Gemelli (FPG) IRCCS, Rome, Italy; 6 Department of Surgical and Medical Sciences, University of Bologna, Bologna, Italy

**Keywords:** MITF, E318K, Melanoma, Renal Cancer, Cancer Risk

## Abstract

The *MITF* E318K variant has been associated with melanoma risk, while risk associated with other variants or of other cancers remains uncertain. We performed a systematic review with meta-analysis of 11 retrospective case-control studies to evaluate cancer risks associated with germline *MITF* variants. Across 8,606 melanoma patients and 17,953 controls, the E318K variant was detected in 2.1% and 0.8% of individuals, respectively, corresponding to a significantly increased melanoma risk (OR 2.55, 95% CI 1.90–3.43). The association was stronger in patients with multiple primary melanomas, with carrier frequencies up to 2.6% compared to 1.0% in single melanoma cases and ORs reaching 4.45 in individual studies. Phenotypic analyses showed enrichment of high nevus burden (> 200 nevi), with ORs up to 12.4 in multiple melanoma cohorts. No consistent association with age at onset or pigmentary traits was observed. Evidence for non-melanoma cancers was limited and heterogeneous: a single study reported increased renal cancer risk (OR 7.64), whereas larger cohorts failed to replicate this finding; an association with pheochromocytoma/paraganglioma was observed (OR 3.19) but lacks confirmation. No other *MITF* variants demonstrated significant cancer risk. These findings support MITF *E318K* as a moderate-penetrance melanoma susceptibility allele, particularly associated with multiple primary melanomas.

**Supplementary Information:**

The online version contains supplementary material available at https://doi.org/10.1007/s10689-026-00603-x.

## Introduction

The *Microphthalmia‑associated Transcription Factor* (*MITF*) codes for a basic helix–loop–helix leucine zipper (bHLH‑Zip) transcription factor that plays a central role in cell survival, differentiation, proliferation, invasion, senescence, metabolism, and DNA damage repair [1]. By regulating genes involved in melanogenesis, cellular metabolism, DNA repair, and stress responses, MITF acts as a master regulator of melanocytic homeostasis [2].

Germline alterations in the *MITF* gene (mapped to chromosome 3p13) are classically associated with Waardenburg syndrome type 2 and Tietz syndrome, both characterized by sensorineural hearing loss and pigmentary abnormalities[3]. Further, the *MITF* gene has been incorporated into diagnostic multigene panels for hereditary cancer predisposition following the demonstration of an association between the E318K variant (exon 10) and an increased risk of cutaneous melanoma and renal cell carcinoma (RCC) [4–5]. Subsequent studies have suggested that carriers may benefit from tailored clinical management strategies, including enhanced dermatologic surveillance and renal imaging protocols [6–8]. Despite these recommendations, the magnitude of cancer risk associated with *MITF* E318K remains incompletely defined, with variability across study designs, populations, and ascertainment criteria, and it remains unknown whether other *MITF* variants confer the same risks.

In this context, the primary objective of the present review was to systematically identify and synthesize the available evidence on cancer risks associated with germline *MITF* variants that can be detected while performing multigene testing, in order to support clinical interpretation and management, as well as to inform future research by highlighting knowledge gaps.

## Materials and methods

### Study design

The study was designed according to the Guidelines of Preferred Reporting Items for Systematic Reviews and Meta-Analysis (PRISMA) Protocol Statement to reduce reporting bias [9]. The main steps included search methods, study selection, quality assessment, data extraction, and data synthesis. A Microsoft Excel-based method [10] was used to record and select data in subsequent steps to promote transparency. The protocol was registered on the Open Science Framework (OSF, https://osf.io/3f5pq).

### Review questions

The question to respond to, formulated according to the PEO (Population, Exposure, Outcomes) methodology (Supplementary Table 1), was: *“What are the cancer risks associated with constitutional pathogenic variants of the MITF gene?”*

### Search strategy and study selection

Four databases (Pubmed, Scopus, Web of Science and Cochrane) were searched for papers published from January 2011 to July 2025. We chose to start in 2011 because *MITF* has been proposed as associated with increased cancer risks in that year. A detailed search strategy for all electronic databases is presented in Supplementary Table 2. The reference lists of all included studies were hand-searched for additional relevant reports or key terms. Targeted internet searching using Google Scholar was also performed to find any additional studies of interest. Grey literature was consulted through the OpenGrey Repository (formerly System for Information on Grey Literature in Europe).

All search results were imported into Microsoft Excel to allow their management, and duplicate records were removed before screening. Titles and abstracts were independently screened by two reviewers (LM, GI), followed by full-text assessment of potentially eligible articles. Any disagreements were resolved through discussion with a third reviewer (DT).

The selection process and reasons for exclusion were documented in a PRISMA flow diagram. To ensure transparency and reproducibility, all steps of the search and selection process were recorded in an electronic logbook following the Excel-based systematic review method described by Godino [10].

### Eligibility criteria

Papers were eligible for inclusion in this systematic review if:

- written in English or Italian language;
- published in peer-reviewed journals and reporting original research (using any methods);
- the study sample explicitly included *MITF* carriers;
- it was possible to assess cancer risks from the study data.

Papers were excluded from the review if they were:

- preclinical research studies;
- pharmacology studies;
- abstract;
- case reports or opinion papers;
- books.

### Assessment of methodological quality and risk of bias

Methodological quality and the risk of bias were independently assessed by LM and CD using the validation tools of Kmet et al. and of Hoy et al. [11–12]; any disagreement was discussed until consensus was reached. According to Kmet et al., items concerning methodology and reporting of results were assessed and assigned 0 point (not addressed), 1 point (partially addressed) or 2 points (completely addressed), with total scores reported as percentages; although a minimum score is not enforced for inclusion in a review, 60% is suggested as a reasonable cut-off. As suggested by Hoy et al., a study was considered to have high, moderate or low risk of bias if 3 or less, 4–6 and 7–11 of the assessed items, respectively, were satisfied. Publication bias was assessed using funnel plots.

### Data analysis and synthesis

Two independent researchers (LM, GI) extracted data from the included studies using a pre-defined matrix that considers the following information: author(s) of the study, year of publication, country where the study was conducted, study aim(s), study design, sample size and findings relevant to the review question. The heterogeneity of included studies was determined. A narrative synthesis was conducted to describe the design, implementation, and findings of the studies. Whenever sufficient information was available and data format allowed comparisons, a synthesis of quantitative data through meta-analysis was performed, using germline *MITF* pathogenic variants as event. Data were analyzed using Review Manager software (Cochrane Collaboration, Copenhagen, Denmark). All quantitative syntheses were restricted to dichotomous outcomes. Pooled odds ratios (Ors) with 95% confidence intervals (Cis) were calculated using a random-effects model, in light of the expected clinical and methodological heterogeneity across studies. Statistical heterogeneity was calculated using the χ^2^ test and I^2^ test. I^2^ values of < 25%, 25–75% or > 75% were considered to indicate low, medium, and high heterogeneity, respectively. The influence of each single study on the results was evaluated by removing each study from consideration one at a time.

### *MITF* variants reporting

The mRNA reference sequence used is the NM_000248.4, corresponding to the MANE Plus Clinical transcript.

## Results

### Search results

**Fig. 1:**
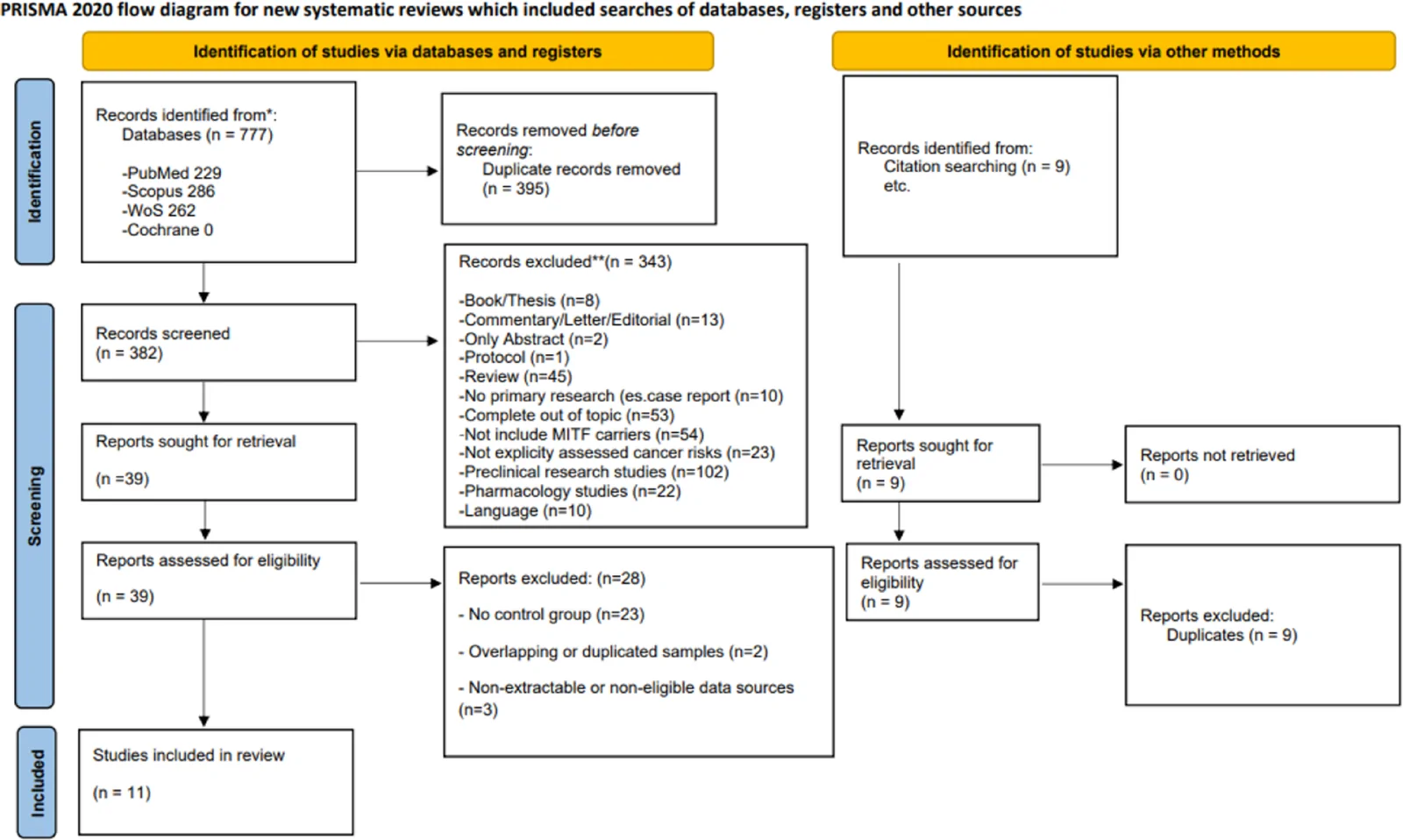
PRISMA 2020 flow diagram of the selection process of the articles

The results of the literature search are summarized in the PRISMA flow diagram (Fig. 1). The database search identified 777 records, of which 395 were removed as duplicates, leaving 382 records for title and abstract screening. Following this initial screening, 39 articles were read in detail by researchers, and 11 met the inclusion criteria and were included in the systematic review.

### Characteristics of included studies

All the 11 included studies [4–5, 13–21] were retrospective series, published between 2011 and 2020, and examined cancer risk associated with *MITF* variants. Two studies were monocentric (one from France and one from Spain), while eight were multicentric (from United States, Canada, Brazil, Australia, Italy, Poland, Switzerland, Denmark). Nine studies focused only on cancer risk associated with the E318K variant, while the other two included the estimation of cancer risks associated with also other two *MITF* variants (c.1081G > A;p.Val320Ile and c.938-325G > A). Eight studies evaluated only melanoma cohorts, while the other three studies evaluated cohorts of different cancer types.

Overall, the risk of bias of the included studies was low and the methodology quality was high. For most studies (10/11, 91.0%) [5, 13–20], data collection, sample characteristics and recruitment were considered appropriate. One study [4] showed a lower methodological quality score (62.5%) and was classified as having a moderate risk of bias. Despite this limitation, the study was retained in the systematic review, as it was the first to report an association between *MITF* variants and cancer risk and provided unique data not available in the other included studies, thereby contributing to a broader interpretation of the evidence.

The main characteristics and findings of the included studies are detailed in Table 1.

**Table 1:** Main characteristics of included studies

Author, date	Country	Population	Disease assessed	Research design	Aim	Main findings	Kmet et al. (2004) score
Bertoletto et al. [5]	France	829 cases, 1659 controls	Melanoma and RCC^a^	Multicentric, Case–control study	To examine E318K MITF prevalence in patients with melanoma and/or renal cell carcinoma (RCC)	The *MITF* E318K variant was associated with an increased risk of melanoma, renal cell carcinoma (RCC), and melanoma plus RCC.	10/16; 62.5%
Berwick et al. 2014 [15]	Australia, Italy, Canada, USA	1194 cases, 2430 controls	Melanoma	Multicentric, Case–control study	To assess E318K effect on melanoma risk and the interaction with other genetic risk factors	The *MITF* E318K variant was associated with an increased risk of melanoma. The association was strongest among individuals with a low‑risk phenotype.	13/16; 81.25%
Castro-Vega et al. 2016 [21]	France	555 cases, 2348 controls	PCC/PGL^b^	Monocentric, Case–control study	To determinate the prevalence of E318K *MITF* in a French cohort of Pheochromocytoma/Paraganglioma (PCC/PGL)	The *MITF* E318K variant was associated with an increased risk of pheochromocytoma/paraganglioma (PCC/PGL).	16/16: 100%
Ghiorzo et al. 2013 [19]	Italy	667 cases, 2205 controls	Melanoma	Multicentric, Case–control study	To test prevalence of E318K *MITF* in 667 Italian melanoma patients	The *MITF* E318K variant was associated with an increased risk of melanoma.	11/16; 68.75%
Gromowsky et al. 2014 [18]	Poland	748 melanoma patients, 683 breast, 753 prostate, 729 colon, 737 lung, 576 kidney, 2114 controls.	Melanoma, breast cancer, prostate cancer, colorectal cancer, lung cancer, kidney cancer	Multicentric, Case–control study	To examine association between E318K and V320I and risk of cancer.	Neither *MITF* E318K nor V320I showed a significant association with melanoma and other cancers.	14/16; 87.5%
Lourenço et al. 2020 [14]	Brazil	247 cases, 280 controls	Melanoma	Multicentric, Case–control study	To identify SNVs on pigmentation-related genes with importance in risk and clinicopathological aspects of CM.	The *MITF* c.938‑325G > A variant did not increase melanoma risk.	15/16; 93.75%
Mangas et al. 2016 [13]	Switzerland	41 cases, 146 controls	Melanoma	Multicentric, Case–control study	To obtain information about genetic predisposition to CM in Ticino.	The *MITF* E318K variant was associated with an increased risk of melanoma.	15/16; 93.75%
McMeniman EK et al. 2019 [20]	Australia	585 patients, 659 controls	Melanoma	Multicentric, Case–control study	To ascertain whether the level of UV damage at the site of melanomas was associated with genetic polymorphisms.	The *MITF* E318K variant was associated with an increased risk of multiple primary melanomas.	15/16; 93.75%
Potrony et al. 2016 [17]	Spain	531 cases, 2704 controls	Melanoma	Monocentric, Case–control study	To evaluate the penetrance of E318K *MITF* in patients with melanoma ad assess association with clinical and phenotypic features.	The *MITF* E318K variant was associated with an increased risk of melanoma.	15/16; 93.75%
Wadt et al. 2015 [16]	Denmark	296 cases (276 CM, 20 μm), 1965 controls	Melanoma (cutaneous and uveal)	Multicentric, Case–control study	To examine frequence of melanoma predisposition gene in patients with cutaneous and uveal melanoma.	The *MITF* E318K variant was associated with an increased risk of melanoma.	12/16; 75.0%
Yokoyama et al. [4]	Australia + UK	3988 patients, 4068 controls	Melanoma	Multicentric, Case–control study	To assess the role of E318K *MITF* variant to developing melanoma.	The *MITF* E318K variant was associated with an increased risk of melanoma.	14/16; 87.5%

a. RCC: renal cells cancer

b. PCC/PGL: Pheochromocytoma/paraganglioma

### Risk and features of melanoma in carriers of the E318K variant in the *MITF *gene

**Fig. 2:**
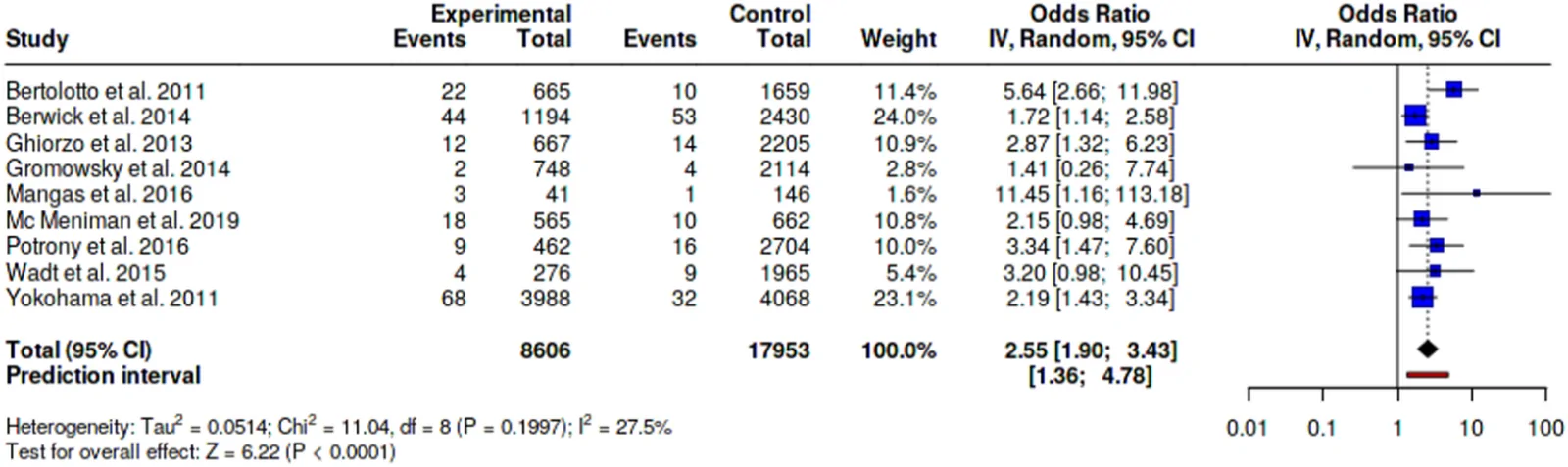
Meta-analysis Odds Ratio of the Association between *MITF* E318K variant and personal history of melanoma

**Fig. 3:**
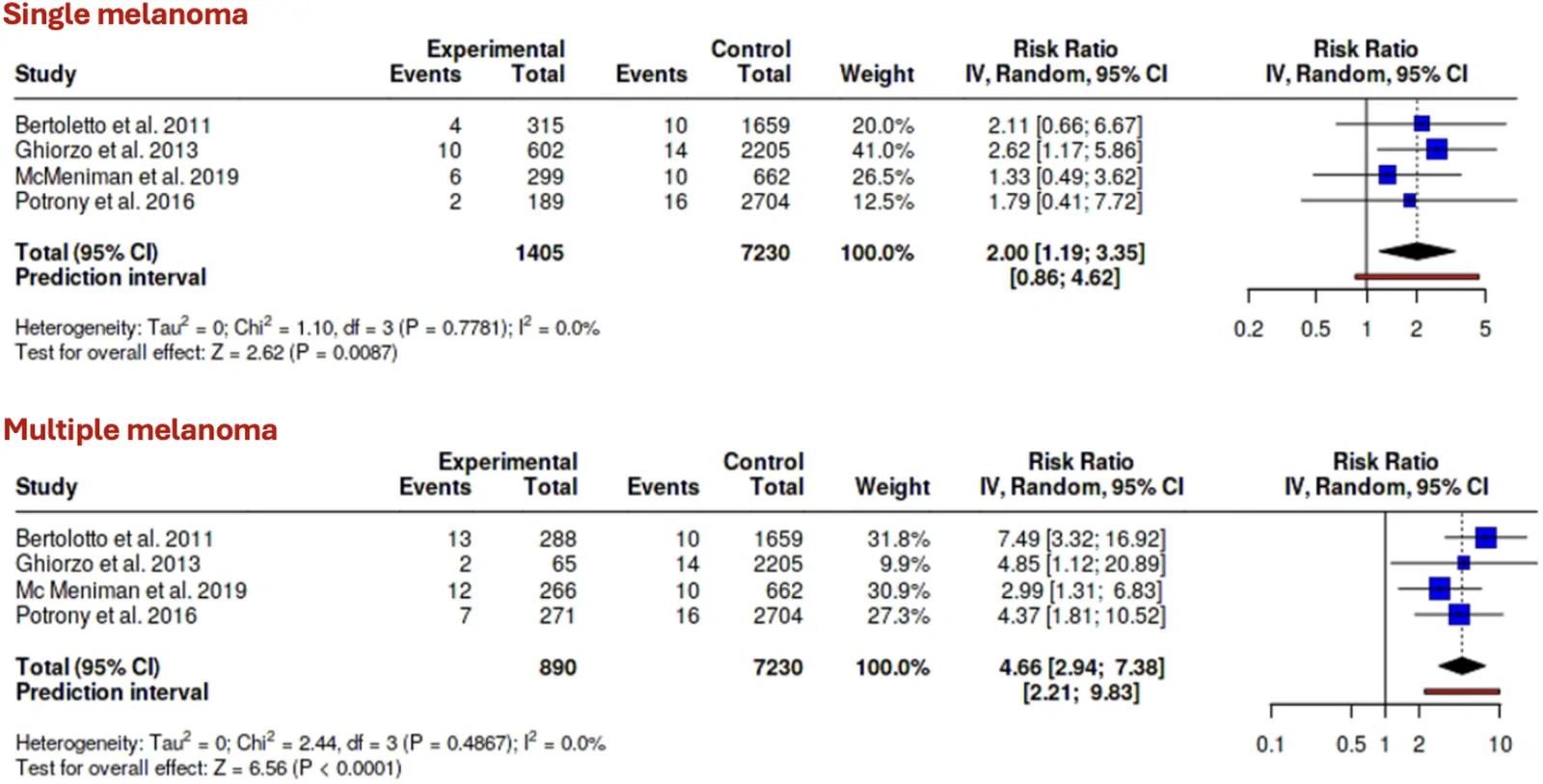
Meta-analysis Odds Ratio of the Association between *MITF* E318K variant and personal history of single and multiple melanoma

A total of 8606 melanoma patients were genotyped for the *MITF* E318K variant, which was detected in 182 patients (2.1%), while 8424 patients (97.9%) were non-carriers (*MITF* E318K wild-type). Out of 17,953 controls, 149 (0.8%) were E318K carriers. Data were subjected to meta-analysis to assess the likelihood of developing melanoma in *MITF* E318K variant carriers: the presence of the variant was associated with an increased risk of melanoma (OR 2.55, CI 1.90–3.43) (Fig. 2 and Supplementary Table 3). Among papers that specify the number of melanomas occurred in individual patients [5, 17, 19–20], the risk of developing multiple primary melanomas (MPMs) results higher than that for single melanomas (Fig. 3 and Supplementary Table 3).

Three studies [4, 15, 17] assessed the relationship between the *MITF* E318K variant and melanoma-related phenotypes (Supplementary Table 4). The variant showed a strong association with very high nevus counts (> 200) in two studies (Potrony et al.: OR 8.4 in all patients and OR 12.4 in MPM cases; Yokohama et al.: OR 2.54), whereas Berwick et al. reported an opposite pattern, linking the variant to the absence of nevi (OR 5.9, CI 1.9–18.0). Regarding pigmentary traits, in the same studies, *MITF* E318K carriers with non-blue eyes appeared to have a higher melanoma risk, although these associations were not statistically significant. Age at melanoma onset, available for 89 carriers across four studies [4, 5, 13, 19], was evenly distributed, with roughly half diagnosed at ≤ 50 years and half after 50.

Data on histological subtypes were available for 45 patients from three studies [5, 17, 19]. Superficial spreading melanoma (SSM) was the most frequently observed subtype, reported in 30 patients (67.0%), followed by nodular melanoma (NM) in 12 cases (27.0%). Other subtypes were less common and included lentigo maligna melanoma (LMM, *n* = 3.7%)), acral melanoma (AM, *n* = 2.4%), and mucosal melanoma (MM, *n* = 1.2%).

### Association of *MITF*E 318K variant with other malignancies

The link between the *MITF* E318K variant and the development of non‑melanoma cancers has been examined across several studies, particularly in relation to renal cancer, pancreatic cancer, and pheochromocytoma/paraganglioma, as summarized in Supplementary Table 5.

Bertolotto et al. documented a substantial increase in RCC risk, reporting more than a sevenfold elevation among carriers who lacked mutations in established RCC‑predisposing genes (OR 7.64; 95% CI 3.15–18.59) [5]. However, other studies did not replicate this association, not confirming that carriers are at increased risk for renal cancer.

Castro‑Vega et al. [21] reported a significant association with pheochromocytoma/paraganglioma after screening 555 unrelated affected individuals (400 with pheochromocytoma, 136 with paraganglioma and 19 with both), observing a higher prevalence of the *MITF* E318K variant (in 5 pheochromocytomas and 2 paragangliomas out of 555 patients − 1.3%) compared with controls (12/2348, 0.5%, OR 3.19; 95% CI 1.34–7.59; *p* = 0.005).

In contrast, Gromowsky et al. [18] found no meaningful correlation between the *MITF* E318K variant and other malignancies, including breast, prostate, colorectal, and lung cancers.

### Association between other *MITF* variants and cancer

To date, no other *MITF* variants have been robustly associated with cancer predisposition with sufficient clinical evidence. Gromowsky et al. [18] genotyped a cohort of 6340 cancer patients, including cases of melanoma, kidney, colorectal, lung, breast, and prostate cancer, for the *MITF* variant c.1081G > A (p.Val320Ile). Only a single carrier of this variant was identified among melanoma patients, while no carriers were observed in any of the other cancer groups.

Similarly, Lourenço et al. [14] investigated the potential role of the intronic *MITF* variant c.938-325G > A, selected from a genome-wide association study (GWAS), in melanoma predisposition. The results did not reach statistical significance (OR 1.28, CI 0.84–1.94; *P* = 0.06), not supporting an association with melanoma risk.

These findings are summarized in Supplementary Table 3.

## Discussion

This systematic review and meta‑analysis provide an updated and comprehensive evaluation of the role of *MITF* germline variants in cancer predisposition. Across 11 retrospective case-control studies published between 2011 and 2020 [4–5, 13–21], the evidence consistently supports a moderate but clinically relevant association between *MITF* E318K and melanoma susceptibility, as already emerged in the meta-analysis study by Guhan et al. [22], while the contribution of other *MITF* variants to cancer risk remains unsubstantiated. The overall methodological quality of the included studies was high, with low risk of bias in most analyses, strengthening the reliability of the conclusions.

The pooled data confirm that *MITF* E318K carriers exhibit an increased likelihood of developing melanoma, with an OR of 2.55 (CI 1.90–3.43). This magnitude of risk aligns with previous observations positioning *MITF* E318K as an intermediate‑penetrance allele, comparable to other moderate‑risk melanoma genes [23]. Notably, the association becomes more pronounced in individuals with multiple primary melanomas (MPMs), for whom the risk nearly doubles. This is in line with previous studies that reported the detection of the *MITF* E318K variant in multiple melanoma patients, as well as the more frequent occurrence of multiple primary melanomas among patients with the variant compared to those without (27% vs. 11%, respectively) [4, 24–25]. This finding reinforces the hypothesis that *MITF* E318K may contribute not only to melanoma initiation but also to a broader phenotype of melanocytic instability, predisposing carriers to recurrent tumorigenesis.

Regarding other clinical features of *MITF* E318K variant carriers, markedly elevated nevus count—particularly > 200 nevi—was consistently associated with the variant in two of the three studies evaluating this phenotype [5, 17]. High nevus burden is a well‑established melanoma risk factor, and its enrichment among carriers may suggest that *MITF* E318K influence melanocyte proliferation or survival. However, the study by Berwick et al. [15] reported an opposite trend, linking the variant to the absence of nevi in MPM patients. This discrepancy may reflect methodological differences, population heterogeneity, or unmeasured environmental modifiers, underscoring the need for standardized phenotypic assessment in future research. However, it could also mean that the *MITF* E318K variant and these clinical features may represent distinct factors that contribute to melanoma development. Pigmentary traits showed weaker and inconsistent associations: non‑blue eye color appeared to modestly increase melanoma risk among carriers although statistical significance was not consistently achieved [5, 15, 17]. Hair and skin color yielded similarly inconclusive results. These findings suggest that the oncogenic effect of *MITF* E318K is not primarily mediated through classical melanoma-associated risk phenotypes, despite the gene’s central role in melanocyte biology. Instead, the variant’s functional impact—disruption of SUMOylation and consequent transcriptional dysregulation—may promote melanoma genesis through mechanisms independent of pigmentation [4, 5]. In addition, in melanocyte models, expression of *MITF* E318K markedly impaired BRAFV600E-induced senescence, as evidenced by reduced β-galactosidase activity, sustained DNA synthesis, and decreased expression of senescence markers such as p16 and p21 [26].

Age at melanoma onset was highly variable [4–5, 13, 19], indicating that *MITF* E318K does not strongly influence the timing of disease presentation. This contrasts with high‑penetrance melanoma genes such as *CDKN2A*, which are typically associated with an early onset of the disease. The balanced age distribution further supports the classification of *MITF* E318K as a moderate‑risk allele with variable expressivity.

Beyond melanoma, the evidence for an association between *MITF* E318K and non‑melanoma malignancies remains heterogeneous. A markedly increased risk of renal cell carcinoma (RCC) was reported only in one study, particularly among individuals lacking mutations in known RCC‑predisposing genes [5]. However, subsequent studies did not replicate this association in sporadic renal cancer cohorts [18], suggesting that the risk cannot be generalized and may be confined to specific genetic or familial contexts. This is also supported by a recent study conducted on large cohorts which analyzed 14,847 cases and 1,700,168 controls and did not document a significantly increased risk of RCC in *MITF* E328K carriers [27].

Regarding other cancers, the association with pheochromocytoma/paraganglioma reported by Castro‑Vega et al. [21] is intriguing and biologically plausible, given the role of *MITF* in neural crest–derived lineages. However, this finding has not yet been replicated, and its clinical significance remains uncertain. Conversely, large‑scale analyses found no evidence linking the variant to other malignancies, suggesting that any broader oncogenic effect is likely absent [18, 28].

Importantly, no other *MITF* variants demonstrated a convincing association with cancer predisposition. Both the c.1081G > A (p.Val320Ile) variant and the intronic c.938‑325G > A variant failed to show significant enrichment in cancer cohorts, reinforcing the unique relevance of the E318K substitution [14, 18].

Taken together, these evidences support *MITF* E318K as a moderate‑penetrance melanoma susceptibility allele with a particularly strong association with multiple primary melanomas and high nevus burden.

Several limitations of the available literature should be acknowledged. First, the included studies differed in terms of study design, cohort composition, ascertainment strategies, and analytical approaches, which limits the comparability of results. Second, although this systematic review was conducted in accordance with rigorous methodological guidelines, it remains possible that some relevant studies were unintentionally missed during the search process, potentially affecting the completeness of the evidence synthesis. Another limitation is the incomplete reporting of demographic characteristics of control populations in the original studies; in particular, age distributions were frequently unavailable, preventing assessment of possible age-related differences between cases and controls that could have influenced the estimated effect sizes. Further, ancestry information was inconsistently reported across studies, preventing evaluation of possible ancestry-specific differences in melanoma risk or age at disease onset; future studies including ethnically diverse populations and standardized reporting of ancestry will be important to clarify whether the clinical impact of *MITF* E318K differs across populations.

Additional case series, ideally based on well‑characterized cohorts with adequate follow‑up, but also searches in large public genomic databases, would be essential to refine current risk estimates and reduce the likelihood that existing data do not accurately reflect the true clinical scenario. Its role in non‑melanoma cancers remains suggestive but not definitive, warranting further investigation into larger, well‑characterized cohorts. Future studies should prioritize standardized phenotyping, exploration of gene–gene and gene–environment interactions, functional analyses to clarify the biological mechanisms underlying the variant’s pleiotropic effects and exploration of the potential interaction of the *MITF* E318K variant with two major melanoma driver mutations, such as those of *BRAF* and/or *NRAS*.

In light of the evidence currently available and acknowledging these limitations, it appears reasonable in clinical practice to exclusively consider the *MITF* E318K variant when evaluating cancer risk in individuals undergoing multigene tumor predisposition panel testing, and to restrict such considerations to melanoma risk. Nevertheless, this approach should be interpreted cautiously and may require revision as additional data become available.

## Conclusion

In conclusion, the available evidence suggests that the *MITF* E318K variant may be regarded as a moderate‑penetrance allele associated with an increased susceptibility to melanoma, particularly for multiple primary melanomas and a high nevus burden. Although emerging data suggest possible associations with selected non‑melanoma malignancies, these findings remain inconsistent and require confirmation in larger, and genetically diverse cohorts. The absence of robust evidence for other *MITF* variants further underscores the unique relevance of the E318K substitution in cancer predisposition. Continued research efforts integrating standardized phenotyping, functional studies, and comprehensive genomic analyses will be essential to clarify the biological mechanisms underlying *MITF*‑related oncogenesis and to refine risk assessment strategies for carriers.

## Supplementary Information

Below is the link to the electronic supplementary material.

Supplementary Material 1

## Data Availability

The data that support the findings of this study are available from the corresponding author upon reasonable request.

## Declarations

### Declarations of generative AI use

The authors confirm that no generative artificial intelligence tools were used in the writing, analysis, or preparation of this manuscript.
