# Supplementary material for: A systematic review of cancer risks associated with MITF variants
Source: Fam Cancer. 2026 Sep 19;25(4):91. doi: 10.1007/s10689-026-00603-x (PMC13589756; doi:10.1007/s10689-026-00603-x)
Supplement: Supplementary file 1 [file 10689_2026_603_MOESM1_ESM.docx]

**SUPPLEMENTARY MATERIAL**

**Supplementary Table 1.** PEO Specifications.

| **Dimension** | **Specification** |
| --- | --- |
| **Population** | Individuals tested for MITF gene |
| **Exposure** | MITF constitutional pathogenic variant |
| **Outcome** | Cancer risks |

| **Supplementary Table 2.** Search strategy. | |
| --- | --- |
| **Electronic database** | **Search terms** |
| PubMed | "MITF"AND (cancer* OR tumor* OR tumour* OR malignanc* OR neoplasm* OR melanoma* OR carcinoma*) AND (risk* OR susceptibil* OR predispos*) |
| Cochrane | "MITF"AND (cancer* OR tumor* OR tumour* OR malignanc* OR neoplasm* OR melanoma* OR carcinoma*) AND (risk* OR susceptibil* OR predispos*) |
| Scopus | "MITF"AND (cancer* OR tumor* OR tumour* OR malignanc* OR neoplasm* OR melanoma* OR carcinoma*) AND (risk* OR susceptibil* OR predispos*) |
| Web of Science | "MITF"AND (cancer* OR tumor* OR tumour* OR malignanc* OR neoplasm* OR melanoma* OR carcinoma*) AND (risk* OR susceptibil* OR predispos*) |
| Google Scholar | "MITF"AND (cancer* OR tumor* OR tumour* OR malignanc* OR neoplasm* OR melanoma* OR carcinoma*) AND (risk* OR susceptibil* OR predispos*) |

**Supplementary Table 3.** Association between *MITF* variants and Melanoma predisposition.

| **Author, date** | **Variant** | **Melanoma** | | |
| --- | --- | --- | --- | --- |
|  |  | **Cases Frequency** | **Controls Frequency** | **OR (CI)** |
| **Bertoletto et al. 2011** | E318K | 22/665 (3.3%) | 10/1659 (0.6 %) | 5.64 (2.66-11.98) |
|  |  | 4/315 (1.2%) [SM] |  | 2.12 [0.66; 6.81] |
|  |  | 13/288 (3.3%)  [MPMs] |  | 7.80 [3.38; 17.95] |
| **Berwick et al. 2014** | E318K | 44/1194 (3.7%) | 53/2430 (2.2%) | 1.7 (1.1-2.6) |
| **Ghiorzo et al. 2013** | E318K | 12/667 (1.8%) [SM] | 14/2205 (0.6%) | 2.87 (1.32-6.23) |
|  |  | 10/602 (2.2%) [MPMs] |  | 2.12 [0.66-6.81] |
|  |  | 2/65 (1.8%) |  | 4.97 [1.11; 22.32] |
| **Gromowsky et al. 2014** | E318K | 2/748 (0.3%) | 4/2114 (0.09%) | 1.41 (0.26-7.74) |
| **Gromowsky et al. 2014** | V320I | 1/748 (0.13%) | 3/2114 (0.14%) | 0.94 (0.10-9.07) |
| **Lourenço et al. 2020** | c.938-325G>A | (48.0%) | (38.6%) | 1.28 (0.84-1.94) |
| **Mangas et al. 2016** | E318K | 3/41 (7%) | 1/146 (0.7%) | 11.45 (1.16–113.18) |
| **McMeniman EK, et al. 2019** | E318K | 18/565 (3.1%) [SM] | 10/662(1.5%) | 2.15 (0.98-4.69) |
|  |  | 6/299 (2.1%) [MPMs] |  | 1.34 (0.48-3.71) |
|  |  | 12/266 (4.5%) |  | 3.08 (1.31-7.22) |
| **Potrony et al. 2016** | E318K | 9/462 (1.9%) | 16/2704 (0.6%) | 4.94 (1.06-22.97) |
|  |  | 2/191 (1.0%) [SM] |  | 1.80 (0.41-7.87) |
|  |  | 7/271 (2.58%) [MPMs] |  | 4.45 (1.82-10.93) |
| **Wadt et al. 2015** | E318K | 4/276 (1.4%) | 9/1965 (0.4%) | 3.20 (0.98-10.45) |
| **Yokoyama et al. 2011** | E318K | 34/2059 (AU)  34/1929 (UK)  68/3988 (TOT)[1.7%] | 14/1953 (AU)  18/2115 (UK)  32/4068(TOT)[0.8%] | 2.33 (1.21-4.70) AU  2.09 (1.14-3.94) UK  2.19 (1.41-3.45) TOT |

**Supplementary Table 4.** Frequency of phenotypic features in melanoma patients according to *MITF* status.

| **Author, date** | **High nevi count** | | | **Non blue eyes** | | | **Fair skin color** | | | **Dark hair color** | | |
| --- | --- | --- | --- | --- | --- | --- | --- | --- | --- | --- | --- | --- |
|  | **Carrier frequency** | **Noncarriers frequency** | **Odds ratio** | **Carrier frequency** | **Non-carriers frequency** | **Odds ratio** | **Carrier frequency** | **Non carriers frequency** | **Odds ratio** | **Carrier frequency** | **Non carriers frequency** | **Odds ratio** |
| Berwick et al 2014 (MPM) | 3/78 (3.8%) | 4/118 (3.4%) | 1.2 (0.2-7.0) | 32/650 (4.9%) | 36/1426 (2.5%) | 2.1 (1.2-3.5) | 30/823 (3.6%) | 30/1627 (1.8%) | 2.2 (1.2-3.8) | 13/304 (4.3%) | 11/765 (1.4%) | OR 3.8 (1.5-9.6) |
| Potrony et al. 2016 | 4/9 (44.4%) | 28/453 (8.7%) | 8.4 (2.14-33.19) | 8/9 (88.9%) | 264/453 (78.3%) | 2.21 (0.27-17.98) | 6/9 (66.7%) | 207/264 (57%) | 1.49 (0.37-6.04) | 6/9 (66.7%) | 232/453 (68.6%) | 0.90 (0.22-3.72) |
| Potrony et al. 2016  (MPM) | 4/7 (57.1%) | 19/264 (9.7%) | 12.4 (2.58-59.7) | 6/7 (85.7%) | 140/264 (73.7%) | 2.1 (0.25-18.24) | 5/7 (71.4%) | 121/264 (58.5%) | 1.8 (0.34-9.37) | 5/7 (71.4%) | 126/264 (65.6%) | 1.3 (0.25-6.93) |
| Yokohama et al. 2011 | n.a. | n.a. | 2.54 (1.42-4.55) | n.a. | n.a. | 2.01 (1.11-3.81) | n.a. | | | n.a. | | |

**Supplementary Table 5.** Association between *MITF* variants and other tumors predisposition.

| **Author, date** | **Variant** | **Other tumors** | | | |
| --- | --- | --- | --- | --- | --- |
|  |  | **Tumor**  **type** | **Cases Frequency** | **Controls Frequency** | **OR (CI)** |
| **Bertoletto et al. 2011** | E318K | Renal cancer | 10/226 (4.6%) | 10/1659 (0.6%) | 7.64 (3.15 – 18.59) |
| **Castro-Vega et al. 2016** | E318K | PCC/PGL | 7/555 (1.3%) | 12/2348 (0.5%) | 3.19 (1.34-7.59) |
| **Gromowsky et al. 2014** | E318K | Renal Cancer | 1/576 (0.17%) | 4/2114 (0.19%) | OR 1.09 (P = 0.64) |
|  |  | Breast Cancer | 1/683 (0.14%) | 4/2114 (0.19%) | OR 1.29 (P = 0.77) |
|  |  | Prostate Cancer | 0/753 | 4/2114 (0.19%) | No carriers |
|  |  | CRC | 1/729 (0.13%) | 4/2114 (0.19%) | OR 1.38 (P = 0.82) |
|  |  | Lung Cancer | 0/737 | 4/2114 (0.19%) | No carriers |
| **Gromowsky et al. 2014** | V320I | Renal Cancer | 0/5760 | 3/2114 (0.14%) | No carriers |
|  |  | Breast Cancer | 0/683 | 3/2114 (0.14%) | No carriers |
|  |  | Prostate Cancer | 0/753 | 3/2114 (0.14%) | No carriers |
|  |  | CRC | 0/729 | 3/2114 (0.14%) | No carriers |
|  |  | Lung Cancer | 0/737 | 3/2114 (0.14%) | No carriers |
